# Abdominal damage control surgery and reconstruction: world society of emergency surgery position paper

**DOI:** 10.1186/1749-7922-8-53

**Published:** 2013-12-17

**Authors:** Laura Godat, Leslie Kobayashi, Todd Costantini, Raul Coimbra

**Affiliations:** 1 Division of Trauma, Surgical Critical Care, and Burns, University of California, San Diego, 200 West Arbor Dr., #8896, San Diego CA 92103-8896, United States of America

**Keywords:** Trauma, Damage control, Abdominal compartment syndrome, Temporary abdominal closure

## Abstract

Damage control laparotomy was first described by Dr. Harlan Stone in 1983 when he suggested that patients with severe trauma should have their primary procedures abbreviated when coagulopathy was encountered. He recommended temporizing patients with abdominal packing and temporary closure to allow restoration of normal physiology prior to returning to the operating room for definitive repair. The term damage control in the trauma setting was coined by Rotondo et al., in 1993. Studies in subsequent years have validated this technique by demonstrating decreased mortality and immediate post-operative complications. The indications for damage control laparotomy have evolved to encompass abdominal compartment syndrome, abdominal sepsis, vascular and acute care surgery cases. The perioperative critical care provided to these patients, including sedation, paralysis, nutrition, and fluid management strategies may improve closure rates and recovery. In the rare cases of inability to primarily close the abdomen, there are a number of reconstructive strategies that may be used in the acute and chronic phases of abdominal closure.

## Introduction

The bloody lethal triad of hypothermia, acidosis, and coagulopathy has been the nemesis of trauma surgeons for decades. Many advances in the field of trauma have evolved around prevention and treatment of this clinical scenario. One useful technique is damage control laparotomy (DCL). DCL has 3 stages, an abbreviated initial operative procedure with temporary abdominal closure (TAC); continued resuscitation and management of physiologic and acid–base derangements, and definitive treatment and closure.

The first stage in DCL is control of hemorrhage and contamination followed by use of a TAC strategy [1]. The optimal TAC strategy should prevent evisceration, evacuate fluid, allow access to the abdominal cavity, and allow for expansion in order to prevent abdominal compartment syndrome (ACS) [2-4]. The second stage of DCL involves continuation of resuscitation, which should include judicious fluid administration with aggressive correction of coagulopathy, acidosis, and hypothermia. Additional management may include paralysis, early enteral nutrition, and diuresis. Lastly, once normal physiology has been restored, the patient should return to the operating room for definitive repair of injuries, followed by abdominal wall closure with reconstruction if possible in the same or in subsequent operative interventions.

DCL has been associated with improved outcomes and decreased mortality in severely injured trauma patients [5,6]. Because of this, DCL indications have been expanded to include abdominal sepsis, ACS, and prolonged or extensive elective surgery. This is a review of the current literature on DCL including recommendations regarding the indications for DCL, techniques of TAC, intensive care unit (ICU) management, and abdominal closure with reconstruction.

To our knowledge no randomized controlled trials (RCT) exist for the use of DCL, although there are many retrospective reviews and prospective observational trials demonstrating improved outcomes in both trauma and acute care surgery populations [2,7].

## Review

### Indications

The decision to switch from definitive treatment to damage control should be made early, ideally prior to entering the operative suite, as this has been associated with improved mortality [7]. In trauma patients, relative pre-operative indications for DCL include systolic blood pressure (SBP) <90 mmHg with penetrating torso, blunt abdominal, or severe pelvic trauma, and the need for resuscitative thoracotomy [1]. Other Emergency Department (ED) variables associated with increased use of DCL include SBP <60 mmHg, hypothermia, inappropriate bradycardia, and pH of <7.2 [8,9]. Intraoperative indications for DCL in trauma patients include “non-surgical” bleeding, pH ≤ 7.18, temperature ≤33°C, transfusion of ≥10 units of blood, total fluid replacement >12 L, and estimated blood losses of ≥5 L [5,6]. Platelet count, PT, aPTT, fibrinogen levels and thromboelastography findings can also be used to guide decision making if available [8].

In addition to the above indications, patients at high risk for ACS should be left open prophylactically at the time of laparotomy [10,11]. This includes patients requiring large volume resuscitation (>15 L or 10 Units of PRBCs), those with evidence of visceral edema, peak inspiratory pressures >40, or intra-abdominal pressure (IAP) >21 during attempted closure [12-16]. Patients with IAP >12 mmHg are considered to have intra-abdominal hypertension (IAH) which is graded from I to IV (Table 1). ACS is a syndrome of organ dysfunction; cardiac, renal or pulmonary associated with elevated IAP and reduced intra-abdominal blood flow [17]. If organ failure has developed patients require emergent decompressive laparotomy or revision of their TAC [12,13,17].

**Table 1:** Grades of intra-abdominal hypertension

**Grade**	*IAP	**Organ failure**
I	12-15	Absent
II	16-20	Absent
III	21-25	Absent
IV	>25	Absent
**ACS	>20	Present

*IAP = Intra-abdominal pressure.

**ACS = Abdominal Compartment Syndrome.

DCL has also been beneficial in general surgery patients with severe abdominal sepsis, including those with diverticulitis or necrotizing pancreatitis who require serial debridement as well as those with significant blood loss [12,18-22]. Patients with mesenteric ischemia or venous occlusive disease who require staged laparotomies due to questionable bowel viability may also benefit from DCL [23]. Advanced age is not a contraindication to DCL as good outcomes have been seen in the elderly [24,25].

Despite improvements in mortality seen in severely injured patients treated with DCL, there is evidence to suggest that it may worsen outcomes in patients who do not meet the indications described above [26]. A retrospective review of over 600 cases, found that low risk patients, identified as those with absence of shock, severe head or combined abdominal injury (Abbreviated Injury Scale <3) had significantly higher rates of infections, organ failure, pulmonary and bowel related complications compared to similar patients closed at the time of their first procedure [27].

### Temporary abdominal closure methods

Because the abdomen is left open at DCL, the resultant wound requires a dressing or TAC. The ideal TAC should be easily and quickly applied, allow room for expansion, limit contamination, decrease bowel edema, protect the viscera, fascia and skin from damage, evacuate fluids, prevent adhesions, minimize loss of domain and be cost effective. The TAC should be easily changed, result in a high rate of closure and be associated with a low rate of complications, particularly enterocutaneous fistula (EC fistula) and mortality (Table 2).

**Table 2:** Methods of temporary abdominal closure (TAC)

**Method of TAC**	**Primary closure rate**	**Mortality rate**	**Enterocutaneous fistula rate**
Bogota Bag/Silo [14,31-36]	12.2-82%	19-58.4%	0-14.4%
Mesh/Wittman Patch [19,42,51,54,55,58]	18-93%	7.7-43%	0-26%
Vacuum Assisted Closure Device [38,39,41,44,45]	31-100%	14-44%	1.2-15%

The first series of DCLs used towel clips or running sutures for closure of the skin or fascia to provide a tamponade effect with peritoneal packing [5]. However, this type of closure frequently resulted in ACS [2,14,28,29], and it is no longer recommended. The next generation TACs were performed using a silo or Bogota bag where a non-permeable barrier; IV bag, bowel bag, steri-Drape or silastic cloth was sutured to the skin or fascia. Advantages are prevention of desiccation, swift application, ability to visualize the bowel and low cost. However, disadvantages include damage to the skin, loss of domain, and lack of effective fluid removal [2,30]. Primary closure rates vary from 12.2-82% [31,32]. EC fistula rates are generally low, reported at 0–14.4% [14,31-36] however ACS rates range as high as 33% [11,33,36]. This method has also largely been abandoned.

Vacuum assisted closure (VAC) devices are most commonly used today. Barker et al., coined the term “vacuum pack” (VP) in 1995; describing a 3 layer TAC; consisting of a fenestrated polyethylene sheet between the abdominal viscera and parietal peritoneum, followed by a moist towel with closed suction drains covered with an occlusive adhesive drape [37]. This method is inexpensive, easily applied and changed, protects the viscera, prevents adhesions, removes exudate and prevents some loss of domain [2,37]. Commercially prepared negative pressure dressings are available and function similar to the VP. These are the V.A.C.©Abdominal Dressing system and the Abthera™ system. Both devices use three layers. The inner layer is a plastic covered sponge that is inserted into the gutters to protect the viscera and facilitate fluid removal, this is followed by a Micro or Macroporous sponge covered by an occlusive dressing that is attached to suction [38-40]. These techniques have been associated with a 31-100% primary closure rate [38-42]. EC fistula rates vary in the literature from 1.2%-15% [41-45], but are generally low. A prospective comparison of these two systems showed higher 30-day primary fascial closure rates and lower 30-day all-cause mortality with the Abthera™ system compared to the Barker VP [46].

Lastly, there are multiple TACs that interpose a graft material between the fascial edges. This can be absorbable such as vicryl or biologic mesh, non-absorbable such as polypropylene (PPE) or expanded polytetrafluoroethylene (ePTFE), or a Wittman patch. The material is initially applied loosely to allow for bowel expansion and prevent ACS. Serial examinations of the wound at the bedside or in the operating room must be done and the mesh is pleated or refastened to gradually pull the fascial edges together [47-49]. The primary benefit of these systems is their ability to maintain and recover fascial domain. Drawbacks include damage to the fascia, inability to prevent adhesions and difficulty with fluid management. EC fistula rates vary with type of graft material; as high as 7-26% with non-absorbable mesh [42,50-52], followed by 4.6-18% with absorbable mesh [49,53,54], and the Wittman patch which has the lowest reported rates of 0–4.2% [55-58]. Risk of ECF is reduced if omentum is interposed between the mesh and bowel [52]. Primary closure has been reported as late as >50 days after the initial damage control operation [49]. ACS rates associated with interposition grafts are seldom sited in the literature; most that did reported no incidences [48,53,54].

### Resuscitation

The second stage of DCL is resuscitation focused on correction of physiologic derangements, acidosis, oxygen debt, coagulopathy and hypothermia [1]. Hemodynamic derangements due to hypovolemic shock should be reversed as quickly as possible with volume resuscitation. However, over use of crystalloids can result in third spacing worsening bowel edema, anastomotic leaks, ACS and multi-organ failure [59,60]. Accordingly, the use of massive transfusion protocols (MTP) has been recommended for DCL patients [60-62]. MTP’s advocate using blood transfusion earlier in resuscitation, using blood and blood products instead of crystalloid or colloid, and the infusion of red cells, plasma, and platelets in a 1:1:1 ratio. There is evidence to suggest that MTP’s and use of 1:1:1 transfusion ratios results in lower overall fluid requirements, blood utilization, and possibly improved mortality in patients with massive blood loss, severe injury and severe physiological derangements, such as are encountered in DCL patients [63,64]. In addition, fluid resuscitation should be guided by hemodynamic parameters such as stroke volume variance or pulse pressure differentials and central venous or left atrial pressures. Improved fluid management may decrease the incidence of ACS and promote early fascial closure [28,65,66]. There is also some evidence that the use of hypertonic fluids in the postoperative period may decrease time to primary closure and improve the primary closure rate [67]. Patients should be monitored for development of ACS and if exhibiting symptoms, the TAC should be removed and replaced with a looser device immediately [2].

Prophylactic antibiotics should be administered preoperatively when possible as infection rates increase if given intra or post operatively [68], and duration should be no longer than 24 hours [69].

It has been proposed that neuromuscular blockade (NMB) can help prevent retraction of the fascial edge and improve closure rates. However, the current evidence comparing NMB to simple sedation is equivocal [44,70]. Similarly diuresis is often suggested as a means to decrease bowel edema and facilitate fascial closure once patients have been resuscitated; however, there is no convincing data to suggest use of diuretics improves the rate or time to closure [71].

Nutrition is known to be a key component to the recovery of patients following severe injury. There are no RCT’s of enteral nutrition in patients with an open abdomen; however multiple retrospective reviews and one prospective cohort study demonstrate safety of enteral nutrition within 36 hours to 4 days of DCL [72-75]. Two studies have demonstrated increased rates of fascial closure [72,73], and 3 demonstrated decreased infectious complications [72,73,75] with early enteral nutrition.

### Closure and abdominal wall reconstruction

Initial return to the operating room should occur as soon as normal physiology has been restored and can vary from 6–72 hours from the time of the primary procedure [2]. Patients should also be taken back to the operating room if there is evidence of surgical bleeding concerning for missed or inadequately addressed injury. A survey from the Western Trauma Association found the majority of its members wait approximately 24 hours for first return to the operating room [2]. Once all injuries have been definitively addressed the abdomen should be closed. The American Association for the Surgery of Trauma studied factors contributing to primary closure and found that those who achieved primary closure were more likely to be women, had lower peak airway pressures, an injury severity score <15, lower lactate levels, higher pH, and lower blood loss. Those who were closed primarily also had fewer EC fistula, abscesses, ICU and ventilator days. Interestingly the volume of crystalloid given was <5 L and did not vary between groups. Overall closure rate was 59.1% [76].

A review of the literature suggest a bimodal distribution of patients with TAC, the first are able to be closed within 4–7 days and achieve a high rate of primary closure, the second group have a delayed (20–40 days) and much lower overall rate of closure [77]. Thus, if unable to close the abdomen within 7 days a progressive closure device may be necessary. This can be achieved using multiple devices, one of the most common; the Wittman patch is sewn to the fascial edges and prevents further loss of domain while slowly bringing the fascial edges together. Multiple studies of the Wittman patch have demonstrated a 78-93% fascial closure rate [55-58]. Modifications of the VAC closure with the addition of retention type sutures in the skin and fascia can significantly increase rates of primary closure to 88-100% [38,39], compared to serial VAC changes which have a primary closure rate of 31% [42]. Absorbable mesh can be used similarly to the Wittman patch, stitching it to the fascia and slowly bringing the fascial edges together during serial returns to the operating room as the visceral edema resolves with primary closure rates of 22-38% [42,50,51].

**Figure 1:**
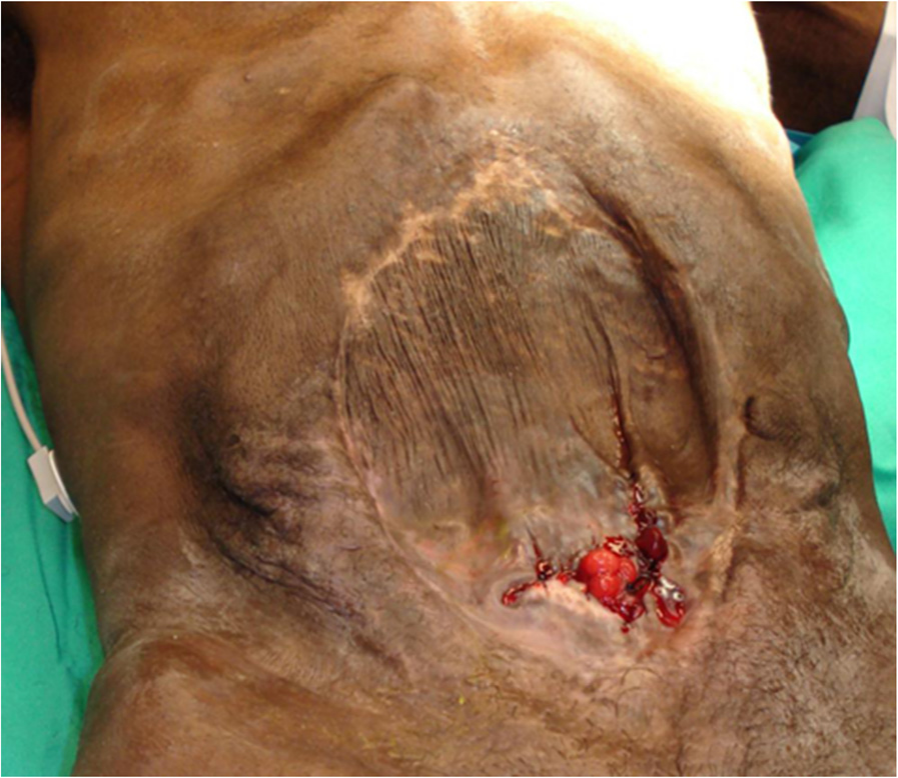
Example of a patient’s abdominal wall with planned ventral hernia after vicryl mesh placement and split thickness skin grafting.

If unable to close the fascial defect with progressive closure techniques, the operative plan must shift gears to one of an expectant hernia (Figure 1). Patients with residual fascial defects should be covered with split thickness skin grafting once the viscera are fixed and granulation tissue is sufficient [42,50,51]. Because of the high risk of infection, synthetic graft material should be removed prior to skin grafting [49].

Formal reconstruction of the ventral hernia should be deferred until after the patient has fully recovered and is ready for another large operation. Timing of the definitive repair is not well studied, Jernigan et al., recommend 6–12 months but no longer as they found less need for prosthetic bridging and lower recurrence rate due to more tension free repair in patients operated on earlier than 12 months. Component separation may be required to span the defect; there are multiple methods for this procedure with good outcomes reported [51]. In clean fields, synthetic mesh may be utilized as a bridge if the patient cannot be closed primarily with or without component separation. Another option to close the fascial defect is to use a biologic material, such as human acellular dermal matrix (HADM). This has the benefit of being an option in a contaminated or infected field. As described by Scott et al., the HADM is fixed transfascially with 2-3 cm of underlay, with multiple pieces stitched together if necessary. The repair should be taut to reduce laxity. If the skin edges can be mobilized and closed, closed suction drains are left to manage the dead space; otherwise a non-adherent dressing is placed over the HADM and a negative pressure dressing is applied [78]. Two series looked at this method [78,79] and reported good outcomes, but with concern for recurrent hernia and eventration.

### Recommendations

#### We recommend

1. Damage control laparotomy for trauma or acute general surgical patients under physiologic stress including; acidosis, hypothermia, hypocoagulable state, prolonged hypotension. Also, those requiring a “second-look” after ischemic or embolic events or intra-abdominal infections which may need additional debridement such as necrotizing pancreatitis.

2. Initial abdominal closure should employ a negative pressure dressing such as the “vacuum pack” method or its commercially available alternative.

3. After 5-7 days if the abdomen cannot be closed convert to the use of a bridging device which progressively brings the fascia together such as the Wittman patch or modified V.A.C.©.

#### We suggest

1. Unless otherwise contraindicated enteral nutrition should be started early.

2. In the absence of definite indication, prophylactic antibiotics should be limited to 24 hours.

3. Formal reconstruction if necessary should be delayed 6-12 months and tempered with a planned ventral hernia.

## Abbreviations

DCL: Damage control laparotomy; TAC: Temporary abdominal closure; ACS: Abdominal compartment syndrome; ICU: Intensive care unit; RCT: Randomized controlled trials; SBP: Systolic blood pressure; ED: Emergency department; PRBCs: Packed red blood cells; IAP: Intra-abdominal pressure; IAH: Intra-abdominal hypertension; EC fistula: Enterocutaneous fistula; VAC: Vacuum assisted closure; VP: Vacuum pack; PPE: Polypropylene; ePTFE: expanded polytetrafluoroethylene; MTP: of massive transfusion protocols; NMB: neuromuscular blockade; HADM: Human acellular dermal matrix.

## Competing interests

The Authors all declare that they have no competing interests.

## Authors’ contributions

All authors helped to draft the manuscript. All authors read and approved the final manuscript.
